# Disparities in access to Dolutegravir in children, adolescents and young adults aged 0–24 years living with HIV in West Africa. A cohort analysis

**DOI:** 10.1101/2024.05.24.24307900

**Published:** 2024-05-25

**Authors:** Sophie Desmonde, Joycelyn Dame, Karen Malateste, Agatha David, Madeleine Amorissani-Folquet, Sylvie N’Gbeche, Mariam Sylla, Elom Takassi, Kouadio Kouakou, Lehila Bagnan Tossa, Caroline Yonaba, Valériane Leroy, IeDEA Pediatric West African cohort.

**Affiliations:** 1. Centre d’Epidémiologie et de Recherche en santé des POPulations (CERPOP), University of Toulouse 3, National Institute for Health and Medical Research (INSERM) UMR 1295Toulouse, France Research Associate; 2. University of Ghana Medical School, and Korle Bu Teaching Hospital, Accra, Ghana* Senior lecturer; 3. University of Bordeaux, National Institute for Health and Medical Research (INSERM) UMR 1219, Research Institute for Sustainable Development (IRD) EMR 271, Bordeaux Population Health Centre, Bordeaux, France; 4. Nigerian Institute of Medical Research (NIMR), Lagos, Nigeria; 5. Cocody University Hospital, Pediatric Department, Abidjan, Cote d’Ivoire; 6. CEPREF Enfant, Abidjan, Côte d’Ivoire; 7. Gabriel Toure Children’s Hospital, Bamako, Mali; 8. Tokoin University Hospital, Lomé, Togo; 9. CIRBA, Abidjan, Côte d’Ivoire; 10. Centre National Hospitalier Universitaire, Cotonou, Benin; 11. CHU Yalgado Ouédraogo, Ouagadougou, Burkina Faso

## Abstract

**Introduction:**

We describe the incidence of Dolutegravir (DTG)-containing antiretroviral treatment (ART) initiation since its introduction in 2019 in pediatric West African IeDEA cohorts.

**Methods:**

We included all patients aged 0–24 years on ART, from nine clinics in Côte d’Ivoire (n=4), Ghana, Nigeria, Mali, Benin, and Burkina Faso. Baseline varied by site and was defined as date of first DTG prescription; patients were followed-up until database closure/death/loss to follow-up (LTFU, no visit ≥ 7 months), whichever came first. We computed the cumulative incidence function for DTG initiation; associated factors were explored in a shared frailty model, accounting for clinic heterogeneity.

**Results:**

Since 2019, 3,350 patients were included; 49% were female;79% had been on ART ≥ 12 months. Median baseline age was 12.9 years (IQR: 9–17). Median follow-up was 14 months (IQR: 7–22). Overall, 1496 (48%) initiated DTG. The overall cumulative incidence of DTG initiation reached 23.6% (95% CI: 21.6–25.6) and 41.3% (95% CI: 39.0–43.6) at 6 and 12 months respectively. Adjusted ART line and available viral load (VL) at baseline, among patients >5 years, DTG-initiation was associated with being male (aHR among 5–9 years: 1.39, 95% CI: 1.06–1.84; among 10–14 years: 1.78, 95% CI: 1.52–2.01; among ≥15 years: 2.46, 95% CI: 2.08–2.91). After 12 months of DTG-implementation, those with detectable VL were less likely to initiate DTG compared to those in viral suppression (aHR: 0.86, 95% CI: 0.76–0.97) and those on NNRTIs were three times more likely to initiate DTG compared to those on PIs (aHR: 3.30, 95% CI: 1.91–5.69).

**Conclusion:**

Children and adolescent males ≥ 5 years, on NNRTIs, with access to VL were the most likely to switch to DTG. As DTG access scales up, updated documentation of treatment practices is required to better ensure universal and equal access.

## INTRODUCTION

Dolutegravir is an integrase strand transfer inhibitor that was first recommended by the World Health Organization (WHO) for antiretroviral therapy (ART) in adults in 2016 [1]. Indeed, DTG-based regimens are promising alternatives, associated with higher viral suppression rates and higher genetic barrier, reducing potential drug resistance [2]. In 2018, the Tsepamo study in Botswana raised concerns about a potential increased risk of neural tube defects in babies born to women who were taking dolutegravir at the time of conception [3]. Subsequently, WHO revised recommendations, advising against the use of DTG among females of reproductive age if reliable contraception measures could not be guaranteed [4]. Further data reported a weaker association between DTG and neural tube defects, and modeling studies supported the use of DTG in all people living with HIV because the benefits outweighed the risk [5–7]. In 2019, the WHO fully recommended dolutegravir-containing ART for all adults and adolescents living with HIV, including females of reproductive age regardless of contraception [8].

However, DTG treatment was inaccessible for most children living with HIV, the only formulation available in Sub-Saharan Africa being the 50mg film-coated tablet registered for use in adults and adolescents weighing > 40kg. Data from the Odyssey trial showed that 50 mg DTG tablets given once daily provide appropriate pharmacokinetic profiles comparable to adults [9,10]. As a result, the WHO 2019 DTG pediatric dosing guidelines led to the US FDA approval of 50mg dosing down to 20kg, widening the pediatric population eligible for DTG [4,11,12]. Odyssey also investigated the dosing of DTG among children receiving rifampicin-containing TB treatment and found twice-daily DTG was safe and sufficient, providing a practical ART option for children with HIV-associated TB [13]. Additional data from the trial, addressed the dosing of DTG in children weighing 3kg to less than 20kg aged 4 weeks and above and formulations are now available for children as young as 4 weeks of age [14].

Since 2019, HIV treatment programs are transitioning from protease inhibitors and non-nucleoside reverse transcriptase inhibitors (NNRTIs), giving priority to those who need it most (1/those not treated, 2/children receiving NNRTI-based regimens, 3/children who need to start TB treatment and 4/children receiving LPV/r solid formulations with challenges in administration and achieving viral suppression) [8,15]. We sought to describe this transition, since 2019, among children and adolescents aged 0–24 years in a large paediatric cohort in West Africa, the IeDEA paediatric West African Database to evaluate AIDS (pWADA).

## METHODS

### Study design and inclusion criteria

The IeDEA paediatric West African Database to evaluate AIDS is aimed to address evolving research questions in the field of HIV/AIDS care and treatment using data from multicentric HIV/AIDS adults and children cohorts in West Africa [16,17]. This collaboration, initiated in July 2006, currently involves 10 paediatric HIV/AIDS clinics in seven countries: Benin (n=1), Burkina Faso (n=1), Côte d’Ivoire (n=4), Ghana (n=1), Mali (n=1), Nigeria (n=1) and Togo (n=1).

We included all children and adolescents aged 0 – 24 years enrolled in an IeDEA pWADA clinic, with at least one visit since July 2019. Those followed up in clinics where DTG was not yet rolled out during the study period were excluded from the analyses.

Each participating country formally agreed to contribute pediatric data, with local institutional review board and National Institute of Health approvals to contribute to the analyses.

### Outcomes and key definitions

Baseline was the date of first documented prescription of DTG in each clinic among patients aged 0–24 years, or date of enrolment, whichever was most recent. Our main outcome was dolutegravir initiation, which we defined as either transitioning to a DTG-containing regimen from another regimen or newly initiating DTG-based ART. Start dates for DTG were based on clinician documentation of a new prescription. Competing events were death or loss-to-program. Loss-to-program included patients with a documented transfer to another clinic or those lost-to-follow-up (LTFU), defined as last clinical contact > 7 months at database closeout date. Database closeout dates varied by site, ranging from 2020–2021; site-specific study periods are available in Table 1.

### Statistical analysis

Patients were followed-up from baseline until DTG initiation, a competing event (death, transfer or LTFU), or database closeout date, when they were censored.

First, we calculated crude cumulative incidence proportions, along with 95% confidence intervals (CIs), for viral suppression and competing events using the Aalen-Johansen estimator. Second, we used multilevel survival analysis to estimate hazard ratios for DTG initiation [18]. We computed a Cox regression model with mixed effects, incorporating a cluster-specific random effect to account for within-cluster homogeneity in outcomes – clusters were clinical sites - and the shared frailty followed a gamma distribution. Covariables included sex, stratified by age at baseline, duration on ART, baseline virological status and ART regimen immediately prior to DTG initiation according to time.

### Patient and public involvement

This study was conducted using programmatic data. Patients were not involved in the analysis plan or result interpretation. Patients did not contribute to the writing or editing of this manuscript.

## RESULTS

Between 2019 – 2021, of the 10 clinical sites included in the pWADA cohort, all except the Sylvanus Olympio Hospital in Togo had initiated DTG roll-out. Documentation of DTG prescription was first in Nigeria (February 2019) and then in Burkina Faso and the Cocody University Hospital in Cote d’Ivoire (March 2019). Other Ivorian sites accessed DTG between May-July 2019, and the clinical sites in Benin and Ghana began roll-out in September 2019. Lastly, the Gabriel Toure Hospital in Mali began prescribing DTG in May 2020.

Overall, 3,350 children and adolescents were included in our study, of whom 3,063 (91.4%) were on ART and in active follow-up at baseline, and 287 (8.6%) ART-naive and newly enrolled during the study period; 47% were female and median age at baseline was 12.5 years (interquartile range [IQR] : 8.4–15.8). Baseline characteristics are presented in table 1. Overall, 59% were of first-line ART, though this varied by clinic : in Benin, Burkina Faso and the CIRBA in Abidjan, >50% were already on second-line regimens, while 80% of patients in Ghana were still on first-line. Overall, 79% had been on ART > 12 months, median time since ART initiation was 6.9 years (IQR : 3.7–9.9). Viral load (VL) at baseline was available for 66% of children, , 59% were in viral suppression (VL< 50 copies/ml). Access to viral load varied across clinical sites and countries, ranging from 33% in Burkina Faso (of whom 48% were in viral suppression) to 91% at the Yopougon University Hospital (of whom 55% were in viral suppression).

**Figure 1 –:**
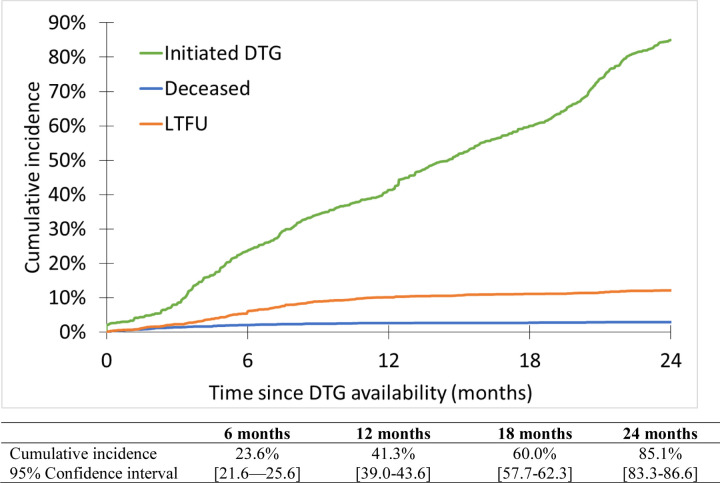
Cumulative incidence of DTG initiation among the 3,350 children, adolescents and young adults living with HIV and enrolled in the IeDEA pediatric West African sites rolling-out DTG

**Figure 2 –:**
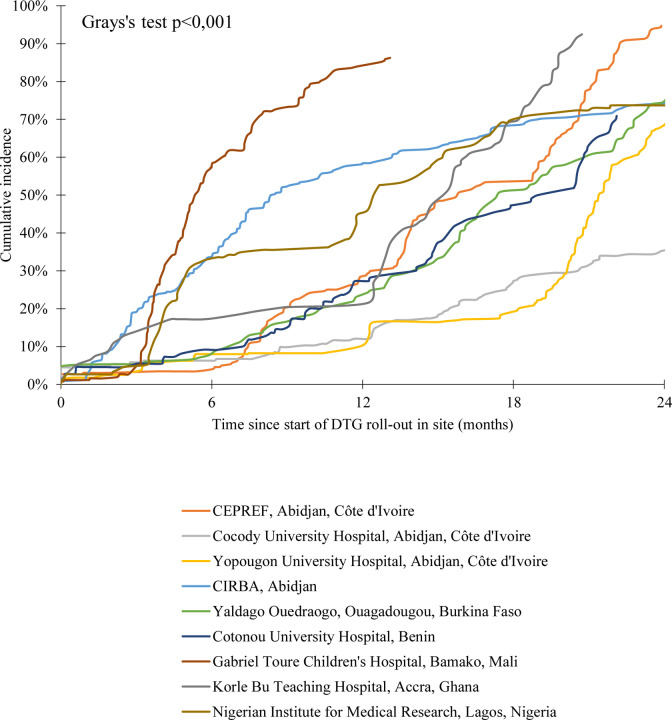
Cumulative incidence of DTG initiation by clinic among the 3,350 children, adolescents and young adults living with HIV and enrolled in the IeDEA pediatric West African sites rolling-out DTG

**Figure 3 –:**
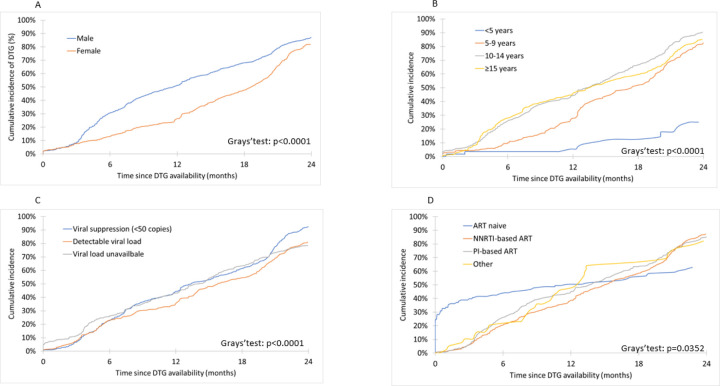
Cumulative incidence of DTG initiation among the 3,350 children, adolescents and young adults living with HIV and enrolled in the IeDEA pediatric West African sites rolling-out DTG by sex (A), baseline age (B), baseline viral load (C) and baseline ART regimen (D).

Overall, 1496 (44.7%) patients initiated a DTG-containing regimen, 50 (1.5%) died and 213 (6.4%) were lost to the program. Median follow-up was 14.0 months [IQR : 7.5–21.9]. Cumulative incidence rates for DTG initiation are described in Figure 1. The 6-month cumulative incidence rate for DTG initiation since roll-out in the clinic was 23.6% (95% Confidence Interval [95%CI] : 21.6–25.6) and reached 41.3% (95%CI : 39.0–43.6) by 12 months. Among those with available follow-up, 24-month incidence of DTG initiation was 85.1% (95%CI : 83.3–86.6). This varied greatly according to region (Figure 2). DTG initiation also varied by sex, with higher cumulative incidence among males compared to females at both 12 and 24 months (Figure 3a). However, we note the gap closing towards the end of the follow-up period.

Factors associated with DTG initiation are presented in table 2. In multivariate analyses, we found that females were less likely to initiate DTG than their male counterpart. This association strongly depended on age, while there was no difference between sex among those aged < 5 years, the adjusted hazard ratio ranged from 1.39 [95%CI : 1.06–1.84] among 5–9 year olds to 2.46 [95%CI : 2.08–2.91] among ≥ 15 olds. Prior ART regimen was also associated with DTG initiation. In the first 12 months since DTG was first prescribed in the clinic, while there was no difference among those on ART, ART-naive children were 4 times more likely to initiate DTG than those on a PI-based ART regimen (aHR : 4.16, 95%CO : 3.10–5.60). After 12 months, DTG initiation was associated with being on an NNRTI-based ART regimen (HR : 2.74, 95%CI : 2.40–3.1) or any other ART regimen (aHR : 2.10, 95%CI : 1.22–3.62) compared to being on PIs; being ART-naive also remained strongly associated (HR : 3.30, 95%CI :1.91–5369).

## DISCUSSION

This study describes DTG roll-out in a large West African cohort of children, adolescents and youth. DTG has been accessible since 2019 in nearly all clinical sites. Transition to DTG-containing ART regimens was less likely in females ≥ 5 years and in those with detectable viral load. Furthermore, we observed a role of previousthe ART regimen at time of DTG roll-out, where those PI-based ART were less likely to access DTG in the later years.

DTG-containing ART regimens were recommended by WHO as preferred first-line ART since 2018 in adults and adolescents [4]. In our cohort, 6 out of the 7 participating countries had began prescribing DTG in 2019, among which all but one site (8/9) began in the first semester of 2019, underlying a rapid scale-up in West Africa.

We found, among children, adolescents and young adults > 5 years, sex disparities, where females were less likely than males of the same age to access DTG since start of the implementation in each clinical site. These disparities increased with age. Although we expected that adolescents of childbearing age might face reduced access to DTG due to early policies and safety communications, this finding highlights a possible lasting impact on DTG uptake among this demographic. Since most sites had begun DTG roll-out before WHO recommended DTG to all, we hypothesise our observation is mostly related to the challenges in DTG access when tied to contraceptive use [3]. Indeed, contraceptive uptake among adolescent girls and young women in Sub-Saharan Africa is low [19,20] and healthcare providers lack education or face bias [21,22]. There is an urgent need to integrate sexual and reproductive health, including contraception and pregnancy planning into adolescent HIV care. We found the gap between males and females in terms of DTG initiation tended to close towards the end of the follow-up period. A similar study in the adult population in West Africa has also reported this trend [23]. We anticipate that as healthcare providers and stakeholders continue to be educated on the evidence of DTG safety as well as sexual and reproductive health among adolescents, access to DTG for adolescent girls and young women will increase and match that of their male counterparts.

Children, adolescents and young adults with detectable viral load were less likely to initiate DTG compared to those in viral suppression. This is contrary to pediatric ARV guidelines recommending that DTG transition should occur irrespective of the availability of viral load test [15]. However, this result is likely driven by the adolescent and young adult population. This is most likely related to anticipated adherence challenges in children and adolescents and inadequate psychological support, which increases the risks of virological failure in this population [24]. Suboptimal adherence to ART has long been identified as a major contributor to the development of drug resistance among people living with HIV [25,26]. Despite the higher genetic barrier in DTG, numerous studies have reported on the emergence of integrase inhibitor drug resistance and reduced efficacy in patients on DTG receiving functional monotherapy [27,28]. However, viral failure in children and adolescents living with perinatally acquired HIV is not necessarily the result of poor adherence but also often pretreatment drug resistance caused by suboptimal maternal ART regimens [29]. But in the absence of drug resistance testing, clinicians are inclined to prioritize therapeutical education, despite persistent challenges in this domain, before transitioning to DTG-containing ART regimens. Affordable technologies for detecting HIV drug resistance among those failing ART are needed to distinguish between children and adolescents living with HIV with HIV drug-resistance and those who have suboptimal adherence. This is important and useful when monitoring patients on DTG, as evidence of pretreatment resistance to DTG in children and adolescents emerge [30].

ART-naive patients were most likely to initiate DTG. This was an expected result since WHO recommendations in the pediatric population prioritize those not treated. Being on a PI-based ART regimen was also associated with DTG initiation. During the first 12-months of DTG roll-out, those on PI-based were more likely to initiate DTG compared to those on NNRTI-based ART. Given that in the earlier years, only the 50mg formulation was available, this observation is likely a result of adolescents and young adults on second-line PI-based ART, being switched to a third-line regimen containing DTG, as per recommendations at the time [4]. In later years, however, we found that those on NNRTI-based ART were 2–3 times more likely to initiate DTG compared to those on PIs. Several reasons can explain this observation. First, for ART-naive children, this was expected since WHO recommendations in the pediatric population prioritize those receiving NNRTI-based regimens [8]. Second, concerns on the development of resistance, as described above, may have led clinicians to favor PI–based regimens if there were anticipated adherence challenges. Indeed, observations from the DAWNING study suggest that failure on PIs is less likely to lead to resistance in adults [31]. Third, countries that had existing stocks of LPV/r pellets or granules were encouraged to consider utilising these stocks, in order to transition to DTG with minimal wastage [32].

We found that children < 10 years were least likely to access DTG. This was an expected result in the early years of our study, since pediatric DTG (pDTG) was not widely availablethen. Cote d’Ivoire, Benin and Nigeria were the first countries to roll-out pediatric formulations [33]. Futhermore, the TORPEDO study, carried out in Benin and Nigeria reported high acceptability and preference for pDTG [34]. However, our results report low transition in the population. While this is most probably explained by the fact that these younger children were on LPV/r, remaining in the system, it may also be a result of poor planification and stock-outs. This is of particular concern in terms of outcomes in these younger children living with HIV, who may remain at high risk of virological failure in the context of slow transition to DTG-containing ART regimens [35]. As pediatric treatment optimization is focused on the continued scale-up of pDTG, irrespective of viral load, and more recently the introduction of pediatric fixed-dose combination of Abacavir-Lamivudine-Dolutegravir (pALD), it is essential for countries to ensure appropriate forecasting and supply, even for small quantities.

Our study presents several limitations. First, the sites involved in these analyses may not be representative of their country. For instance, we reported no DTG roll-out in Togo, although previous literature has reported on the beginning of DTG roll-out since October 2019 [36]. Second, we report that DTG roll-out began in 2019, shortly before the COVID-19 pandemic, where healthcare provision may have been different from that of standard care, thus affecting factors associated with DTG initiation. Third, this was carried in the early years of DTG roll-out, with varying database closeout dates ranging 2021–2022. This was amidst perinatal safety concerns regarding the use of DTG among women of childbearing age and before pDTG was rolled out.

This study however provides useful real-world evidence on the scale-up of DTG in children, adolescents and young adults in West Africa. While we anticipate that sex disparities and the slow transition among younger children may fade in the coming years, access to DTG remains unequal. Lack of viral load and wastage concerns should not be a barrier to DTG initiation. Continued monitoring of DTG implementation and better planification strategies are important to ensure universal access to all.

## Figures and Tables

**Table 1 –:** Baseline characteristics of the 3,350 patients 0–24 years living with HIV and enrolled in the IeDEA pediatric West African sites rolling-out DTG

	CEPREF		CHUC		CHUY		CIRBA		CHUYO		CNHU		GABRIEL		KBTH		NIMR		OVERALL		p-value *
Total	446		396		406		374		243		247		633		305		300		3350		
**Date of DTG introduction**	11th May 2019		27th March 2019		3rd July 2019		7th May 2019		7th March 2019		3rd September 2019		27th May 2020		25th September 2019		22nd February 2019				
**Date of Database closure**	14th June 2021		15th October 2021		18th July 2022		29th March 2022		15th April 2022		8th August 2021		10th July 2021		13th July 2021		5th March 2022				
**Sex**																					0,0518
Male	207	46,4%	209	52,8%	202	49,8%	199	53,2%	124	51,0%	141	57,1%	357	56,4%	170	55,7%	161	53,7%	1770	52,8%	
Female	239	53,6%	187	47,2%	204	50,2%	175	46,8%	119	49,0%	106	42,9%	276	43,6%	135	44,3%	139	46,3%	1580	47,2%	
**Age at baseline**																					<,0001
Median, [IQR]	14,3	[9,8–17,3]	11,7	[7,3–15,2]	14,5	[10,1–17,7]	14,3	[11,2–17,6]	11,1 [8,1–13,9]		12	[7,3–15,2]	13,2	[9,1–16,5]	9,8	6,0–12,7]	10,7	[8,2–12,8]	12,5	[8,4–15,8]	
< 2 years	5	1,1%	24	6,1%	7	1,7%	2	0,5%	8	3,3%	12	4,9%	27	4,3%	16	5,2%	3	1,0%	104	3,1%	
2– 4 years	23	5,2%	33	8,3%	29	7,1%	20	5,3%	19	7,8%	23	9,3%	49	7,7%	34	11,1%	15	5,0%	245	7,3%	
5–9 years	88	19,7%	98	24,7%	63	15,5%	54	14,4%	75	30,9%	57	23,1%	114	18,0%	108	35,4%	111	37,0%	768	22,9%	
10–14 years	133	29,8%	138	34,8%	122	30,0%	135	36,1%	97	39,9%	91	36,8%	210	33,2%	134	43,9%	167	55,7%	1227	36,6%	
>15 years	197	44,2%	103	26,0%	185	45,6%	163	43,6%	44	18,1%	64	25,9%	233	36,8%	13	4,3%	4	1,3%	1006	30,0%	
**ART regimen at baseline**																					<,0001
ART-naive	31	7,0%	76	19,2%	14	3,4%	1	0,3%	34	14,0%	29	11,7%	43	6,8%	48	15,7%	11	3,7%	287	8,6%	
NNRTI-based ART	293	65,7%	243	61,4%	266	65,5%	187	50,0%	113	46,5%	95	38,5%	366	57,8%	241	79,0%	233	77,7%	2037	60,8%	
PI-based ART	122	27,4%	77	19,4%	126	31,0%	159	42,5%	93	38,3%	89	36,0%	224	35,4%	16	5,2%	56	18,7%	962	28,7%	
Other ART regimens	0	0,0%	0	0,0%	0	0,0%	27	7,2%	3	1,2%	34	13,8%	0	0,0%	0	0,0%	0	0,0%	64	1,9%	
**ART line at baseline**																					<,0001
ART naive	31	7,0%	76	19,2%	14	3,4%	1	0,3%	34	14,0%	29	11,7%	43	6,8%	48	15,7%	11	3,7%	287	8,6%	
1st line	301	67,5%	242	61,1%	259	63,8%	169	45,2%	109	44,9%	135	54,7%	291	46,0%	245	80,3%	229	76,3%	1980	59,1%	
≥ 2nd line	114	25,6%	78	19,7%	133	32,8%	204	54,5%	100	41,2%	83	33,6%	299	47,2%	12	3,9%	60	20,0%	1083	32,3%	
**Time on ART**																			0	0,0%	<,0001
ART naive	31	7,0%	76	19,2%	14	3,4%	1	0,3%	34	14,0%	29	11,7%	43	6,8%	48	15,7%	11	3,7%	287	8,6%	
< 12 months	95	21,3%	21	5,3%	5	1,2%	89	23,8%	8	3,3%	28	11,3%	170	26,9%	7	2,3%	0	0,0%	423	12,6%	
≥ 12 months	320	71,7%	299	75,5%	387	95,3%	284	75,9%	201	82,7%	190	76,9%	420	66,4%	250	82,0%	289	96,3%	2640	78,8%	
**In virological success (< 50 copies), among those on ART within 6 months of baseline**																					<,0001
VL available	390	87,4%	300	75,8%	369	90,9%	317	84,8%	80	32,9%	92	37,2%	365	57,7%	120	39,3%	186	62,0%	2219	66,2%	
Success (%of avail)	240	61,5%	173	57,7%	203	55,0%	208	65,6%	38	47,5%	46	50,0%	216	59,2%	55	45,8%	140	75,3%	1319	59,4%	
Failure (%of avail)	150	38,5%	127	42,3%	166	45,0%	109	34,4%	42	52,5%	46	50,0%	149	40,8%	65	54,2%	46	24,7%	900	40,6%	
VL unavailable	56	12,6%	96	24,2%	37	9,1%	57	15,2%	163	67,1%	155	62,8%	268	42,3%	185	60,7%	114	38,0%	1131	33,8%	

**Table 2 -:** Factors associated with transitionning to DTG-containing ART regimen among the 3,350 children, adolescents and young adults living with HIV and enrolled in the IeDEA pediatric West African sites rolling-out DTG

	Adjusted hazard ratio		95% CI		p-value
**Sex**					*0.0022*
< 5 years, male vs female	1.64		[0.55 – 4.90]		
5–9y, male vs female	1.39		[1.06 – 1.84]		
10–14y, male vs female	1.78		[1.52 – 2.01]		
≥ 15y, male vs female	2.46		[2.08 – 2.91]		
**On ART > 12 mths** vs ≤12 mths	1.17		[0.99 – 1.37]		*0.0603*
**Virological status at baseline** (ref: viral suppression)					*0.0573*
Detectable viral load	0.86		[0.76 – 0.97]		
Viral load unavailable	0.94		[0.81 – 1.08]		
**Baseline ART regimen** (ref: PI-based ART)					<*0.0001*
	*Within the first 12 months*		> *12 months of follow-uo*		
ART naive	4.16	[3.10 – 5.60]	3.30	[1.91 – 5.69]	
NNRTI-based ART	0.77	[0.67 – 0.89]	2.74	[2.40 – 3.14]	
Other ART regimens	0.88	[0.54 – 1.44]	2.10	[1.22 – 3.62]	

## Key messages

### What is already known on this topic ?

- Dolutegravir (DTG)-based ART regimens are recommended as preferred first line ART regimen since 2018 in adolescents and 2019 in all children and adolescents.
- Universal DTG access in adults in West Africa has faced challenges such as infrastructure challenges, and healthcare system disparities, and was hindered by early perinatal safety concerns greatly affecting greatly women of childbearing age.
- Specific data in children, adolescents and young adults in West Africa is limited.

### What this study adds ?

- We observed rapid scale-up of DTG among children, adolescents and young adults living with HIV, despite the COVID-19 epidemic.
- DTG access however, remained, unequal, with school-aged and adolescent girls and young women being less likely to initiate DTG, as were those with detectable viral load.
- Younger children, < 5 years were less likely to initiate DTG, explained by the later approval and low availability of pediatric formulations.
- We also found that those on PI-based ART were less likely to transition to DTG, underlying possible remnant ritonavir-boosted lopinavir and wastage concerns.

### How this study might affect research, practice or policy?

- Training and updating guidance for healthcare workers is essential to ensure universal access to DTG, especially for girls, where unequity begins at 5 years.
- Efforts to improve access to DTG in West Africa require multifaceted interventions including healthcare infrastructure improvement and facilitation of pediatric antiretroviral forecasting and planification.
