# Supplementary material for: Disparities in dolutegravir utilisation in children, adolescents and young adults (0–24 years) living with HIV: An analysis of the IeDEA Paediatric West African cohort
Source: medRxiv. 2024 Nov 8:2024.05.24.24307900. Originally published 2024 May 25. Preprint. [Version 3] doi: 10.1101/2024.05.24.24307900 (PMC11142258; doi:10.1101/2024.05.24.24307900)
Supplement: Supplement 1 [file media-1.pdf]

Table S1 - Patient-level baseline characteristics of the 3,350 patients 0-24 years living with HIV and enrolled in the participating IeDEA pediatric West African clinical , 2019-2022

**Country/Clinics**	**Côte d'Ivoire CEPREF**		**Côte d'Ivoire CHU Cocody**		**Côte d'Ivoire CHUYopogon**		**Côte d'Ivoire CIRBA**		**Burkina Faso CHUYO**		**Benin CNHU**		**Mali GABRIEL**		**Ghana KBTH**		**Niegeria NIMR**		***p-value****
**Total**	446		396		406		374		243		247		633		305		300		
**Date of DTG introduction (baseline)**	11th May 2019		27th March 2019		3rd July 2019		7th May 2019		7th March 2019		3rd September 2019		27th May 2020		25th September 2019		22nd February 2019		
**Date of database closure**	14th June 2021		15th October 2021		18th July 2022		29th March 2022		15th April 2022		8th August 2021		10th July 2021		13th July 2021		5th March 2022		
**Number of months of DTG roll-out**	25		31		37		35		37		23		13		22		36		
**Time to 1st visit since DTG introduction, me**	41 [18-74]		22 [9-50]		30 [9-63]		51 [17-126]		126 [61-224]		56 [15-107]		48 [26-71]		49 [26-71]		49 [21-70]		<.0001
**Sex**																			0,0518
Male	207	46.4%	209	52.8%	202	49.8%	199	53.2%	124	51.0%	141	57.1%	357	56.4%	170	55.7%	161	53.7%	
Female	239	53.6%	187	47.2%	204	50.2%	175	46.8%	119	49.0%	106	42.9%	276	43.6%	135	44.3%	139	46.3%	
**Age at baseline**																			<.0001
Median, [IQR]	14.3 [9.8-17.3]		11.7 [7.3-15.2]		14.5 [10.1-17.7]		14.3 [11.2-17.6]		11.1 [8.1-13.9]		12 [7.3-15.2]		13.2 [9.1-16.5]		9.8 6.0-12.7]		10.7 [8.2-12.8]		
< 2 years	5	1.1%	24	6.1%	7	1.7%	2	0.5%	8	3.3%	12	4.9%	27	4.3%	16	5.2%	3	1.0%	
2- 4 years	23	5.2%	33	8.3%	29	7.1%	20	5.3%	19	7.8%	23	9.3%	49	7.7%	34	11.1%	15	5.0%	
5-9 years	88	19.7%	98	24.7%	63	15.5%	54	14.4%	75	30.9%	57	23.1%	114	18.0%	108	35.4%	111	37.0%	
10-14 years	133	29.8%	138	34.8%	122	30.0%	135	36.1%	97	39.9%	91	36.8%	210	33.2%	134	43.9%	167	55.7%	
>15 years	197	44.2%	103	26.0%	185	45.6%	163	43.6%	44	18.1%	64	25.9%	233	36.8%	13	4.3%	4	1.3%	
**ART regimen at baseline**																			<.0001
ART-naive	31	7.0%	76	19.2%	14	3.4%	1	0.3%	34	14.0%	29	11.7%	43	6.8%	48	15.7%	11	3.7%	
NNRTI-based ART	293	65.7%	243	61.4%	266	65.5%	187	50.0%	113	46.5%	95	38.5%	366	57.8%	241	79.0%	233	77.7%	
PI-based ART	122	27.4%	77	19.4%	126	31.0%	159	42.5%	93	38.3%	89	36.0%	224	35.4%	16	5.2%	56	18.7%	
Other ART regimens	0	0.0%	0	0.0%	0	0.0%	27	7.2%	3	1.2%	34	13.8%	0	0.0%	0	0.0%	0	0.0%	
**ART line at baseline**																			<.0001
ART naive	31	7.0%	76	19.2%	14	3.4%	1	0.3%	34	14.0%	29	11.7%	43	6.8%	48	15.7%	11	3.7%	
1st line	301	67.5%	242	61.1%	259	63.8%	169	45.2%	109	44.9%	135	54.7%	291	46.0%	245	80.3%	229	76.3%	
≥ 2nd line	114	25.6%	78	19.7%	133	32.8%	204	54.5%	100	41.2%	83	33.6%	299	47.2%	12	3.9%	60	20.0%	
**Time on ART**																			<.0001
ART naive	31	7.0%	76	19.2%	14	3.4%	1	0.3%	34	14.0%	29	11.7%	43	6.8%	48	15.7%	11	3.7%	
< 12 months	95	21.3%	21	5.3%	5	1.2%	89	23.8%	8	3.3%	28	11.3%	170	26.9%	7	2.3%	0	0.0%	
≥ 12 months	320	71.7%	299	75.5%	387	95.3%	284	75.9%	201	82.7%	190	76.9%	420	66.4%	250	82.0%	289	96.3%	
**Virological status (success : viral load 50 copies).**																			<.0001
Viral load (VL) available	390	87.4%	300	75.8%	369	90.9%	317	84.8%	80	32.9%	92	37.2%	365	57.7%	120	39.3%	186	62.0%	
Success (%of available)	240	61.5%	173	57.7%	203	55.0%	208	65.6%	38	47.5%	46	50.0%	216	59.2%	55	45.8%	140	75.3%	
Failure (%of available)	150	38.5%	127	42.3%	166	45.0%	109	34.4%	42	52.5%	46	50.0%	149	40.8%	65	54.2%	46	24.7%	
