# Supplementary figures and images for: Disparities in dolutegravir utilisation in children, adolescents and young adults (0–24 years) living with HIV: An analysis of the IeDEA Paediatric West African cohort
Source: medRxiv. 2024 Nov 8:2024.05.24.24307900. Originally published 2024 May 25. Preprint. [Version 3] doi: 10.1101/2024.05.24.24307900 (PMC11142258; doi:10.1101/2024.05.24.24307900)

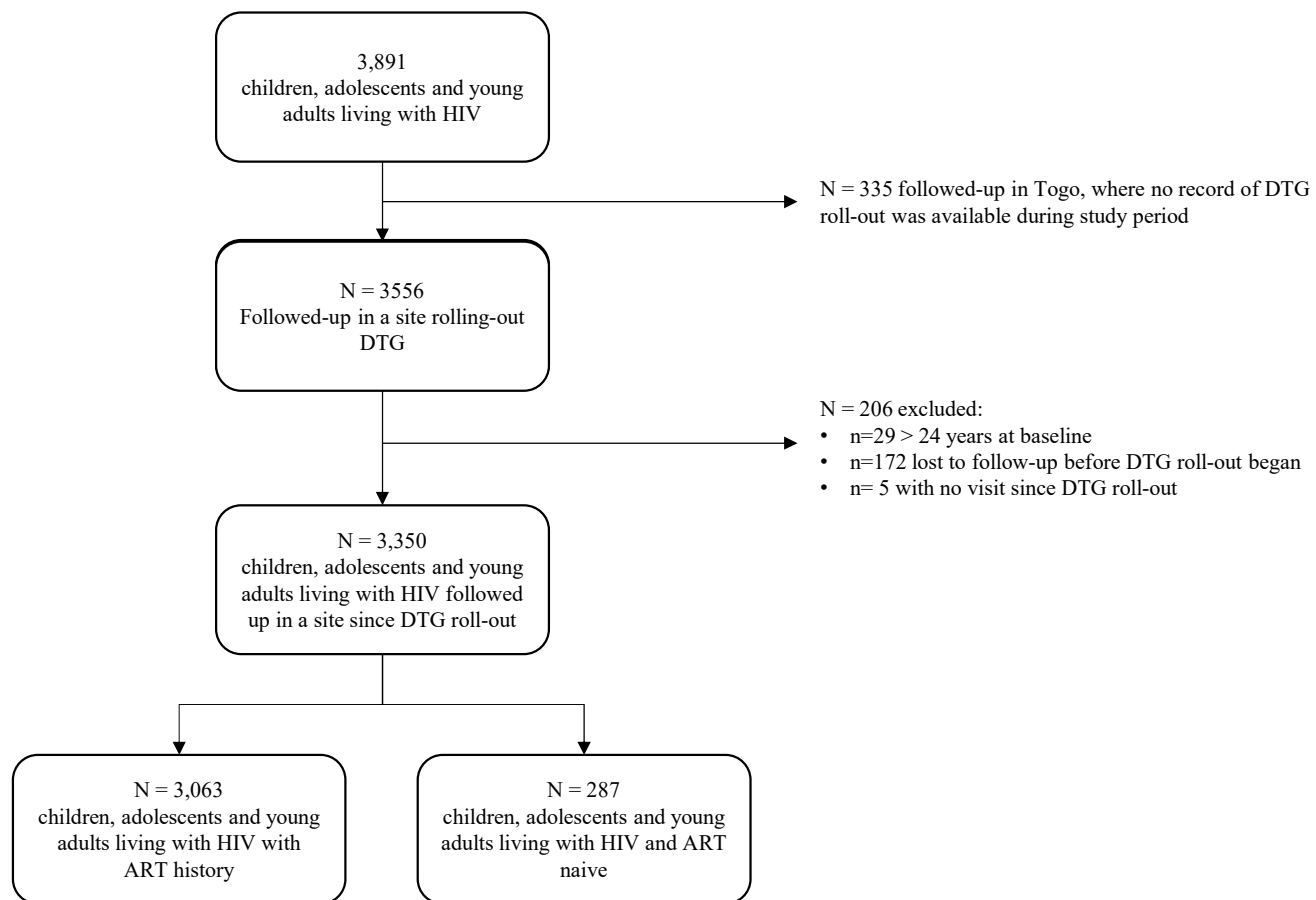

**Figure A** – Patient inclusion flow diagram

Supplement: Supplement 2 [file media-2.pdf]
